# Case Report: Tissue Origin Identification for Cancer of Unknown Primary: Gene Expression Profiling Approach

**DOI:** 10.3389/fonc.2021.702887

**Published:** 2021-11-11

**Authors:** Xingxiang Pu, Sa Yang, Yan Xu, Bolin Chen, Qianzhi Wang, Qian Gong, Lin Wu

**Affiliations:** ^1^ Department of Thoracic Medical Oncology, Hunan Cancer Hospital/The Affiliated Cancer Hospital of Xiangya School of Medicine, Central South University, Changsha, China; ^2^ Department of Pharmacy, Xiangxiang People’s Hospital, Xiangxiang, China; ^3^ Department of Pharmacy, Hunan Cancer Hospital/The Affiliated Cancer Hospital of Xiangya School of Medicine, Central South University, Changsha, China

**Keywords:** cancer of unknown primary, gene expression profiling, tissue of origin, real-time PCR, immunohistochemistry

## Abstract

The treatment of cancer of unknown primary (CUP) is a huge challenge for clinicians. Gene expression profiling can help identify the tissue origin of tumors by detecting the expression levels of specific genes in tumor tissues. Herein, we report four CUP cases. All of them have been successfully identified with the corresponding primary tumor sites through gene expression profiling analysis. Then all patients received accurate treatment, providing reference to guide therapeutic decisions to treat CUP tumors in the future.

## Introduction

Cancer of unknown primary (CUP) is a well-recognized clinical syndrome, in which clinical, radiographic, and pathologic evidence of a primary site of origin is lacking (1). Adenocarcinoma and poorly differentiated carcinoma are the most common tissue types of CUP. As reported to be the malignant tumor with the seventh or eighth highest incidence, CUP accounts for 3–5% of total cancer diagnoses worldwide; and with its short course and rapid progression (2, 3), it is also the fourth most common cause of cancer death (4). Life expectancy statistics largely vary, but one meta-analysis indicated a median survival of only 4.5 months, with a 1-year survival rate of 20% and a 5-year survival rate of 4.7% (5). Therefore, an accurate diagnosis is essential for treatment and prognosis.

Recently, researchers have found that the gene expression profiling (GEP) of metastatic tumors differ from those of tissues at the metastatic site but are similar to those of tissues at the primary site, suggesting that during the process of tumorigenesis, development, and metastasis, the metastatic tumor retains the gene expression characteristics of its tissue of origin. Based on this assumption, Ye et al. developed a real-time PCR (RT-PCR) based assay (the 90-gene expression assay) for identifying the tissue of origin based on GEP analysis (6). Initially, a pan-cancer transcriptome database comprising 5434 samples representing 21 tumor types was established to identify the tumor-specific genes. Next, the Top-10 most predictive genes for each of the 21 tumor types were selected by using the Support Vector Machine Recursive Feature Elimination (SVM-RFE) algorithm. Finally, a list of 90 genes was identified after removing redundant genes and the 90 genes were used for establishing an SVM linear model termed “90-gene classifier” to identify tumor tissue of origin. Intuitively, the similarity scores for each of the 21 tumor types were calculated by the 90-gene classifier, which reflects how much the gene expression pattern of the test specimen is similar to the global gene expression pattern with known tumor type. The similarity scores were probability-based, with a reported range from 0 to 100, and all 21 similarity scores sum to 100. The tumor type with the highest similarity score was considered as the predicted tumor type by the 90-gene classifier. In a multisite study, the performance of the 90-gene expression assay was illustrated in 609 tumor samples of known primary origin, with an accuracy of 89.8%. More specifically, the classification accuracy reached 90.4% in primary tumors and 89.2% in metastatic tumors. Moreover, in a real-world cohort comprising 141 CUP patients, the 90-gene expression assay was able to provide helpful predictions of tumor origin in 71.6% of patients (101 of 141) (6). In the present study, we employed the 90-gene expression assay to identify the primary sites of four CUP cases and evaluated the therapeutic outcomes in these patients after receiving treatment guided by GEP-based source-tracing, aiming to provide referencing information to guide the diagnosis and treatment decisions in other CUP cases.

## Case Report

**Figure 1:**
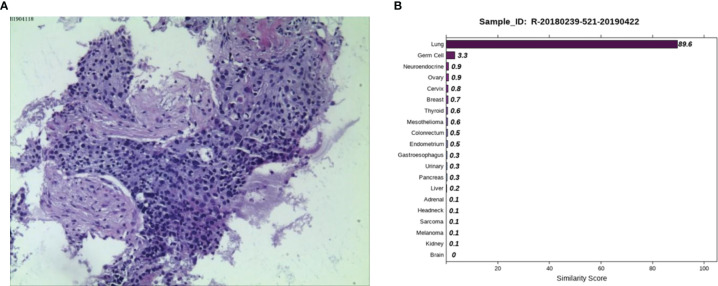
Pathological and 90-gene expression assay results of case 1. **(A)** The pathological diagnosis of the resected specimens was squamous cell carcinoma. Immunohistochemistry staining: P40 (-), Ki67 (90%+), TTF1 (-), CK7 (-), P16 (-). **(B)** The 90-gene expression assay showed that the similarity score of the right lung mass coming from the lung tissue is 89.6.

Case 1: A 45-year-old female was admitted to a tertiary hospital in Xinjiang Province due to vaginal bleeding following three months of sexual activity and had just been diagnosed with cervical cancer on March 10, 2014. Computed tomography (CT) examination revealed cervical cancer and multiple enlarged lymph nodes in the pelvic cavity and bilateral groin area. Histopathological analysis from an outside hospital suggested moderately differentiated cervical squamous cell carcinoma, and interventional treatment and chemotherapy (paclitaxel combined with cisplatin) were administered. Then, laparoscopic extensive uterus, bilateral salpingectomy, pelvic lymph node dissection, and ovarian suspension (bilateral) were implemented under general anesthesia. On May 12, 2014, the postoperative pathological result showed cervical invasive moderately differentiated squamous cell carcinoma with the tumor-infiltrating in nearly all layers of the cervical wall, and tumor cell metastasizing to the lymph node of the left inner iliac (1/1). On June 13, 2014, she was given three-dimensional intensity-modulated radiotherapy in the external pelvic area (DT 4500cGY/25 times) and in abdominal enlarged lymph nodes of the external pelvic area (DT6000cGY/25 times), followed by three cycles of chemotherapy (paclitaxel combined with cisplatin). No obvious signs of recurrence or metastasis were found in regular review after discharge. Reexamination of CT on March 27, 2019, revealed progression in the lower lobes of the lung. A lung biopsy was performed by an outside provider under the guidance of CT, and the pathological findings (**Figure 1A**) were consistent with the previous conclusion of squamous cell carcinoma. Thus, the practitioners considered the possibility of metastatic cervical cancer. The immunohistochemistry (IHC) results suggested P40 (-), Ki-67 (90%+), TTF1 (-), CK7 (-), and P16 (-). Next, she was admitted to Hunan Cancer hospital on April 1, 2019. CT results showed the occupancy of the right lower lung dorsal segment and no obvious abnormality in the abdominal and pelvic cavity. We considered the possibility of primary lung cancer after evaluating the pathological results and related inspections. To further clarify the primary tumor, the 90-gene expression assay was performed on the biopsy tissue of the right lung tumor, and the test results (**Figure 1B**) indicated that the right lung mass was primary lung cancer. Finally, the patient was diagnosed with primary bronchial lung cancer, right lower lobe squamous cell carcinoma and underwent video-assisted thoracic surgery (VATS) radical resection of the right lower lobe cancer under general anesthesia on April 30, 2019 (pT2aN0M0, stage IB). The patient is in a disease-free state after regular reexamination so far without further adjuvant chemotherapy.

**Figure 2:**
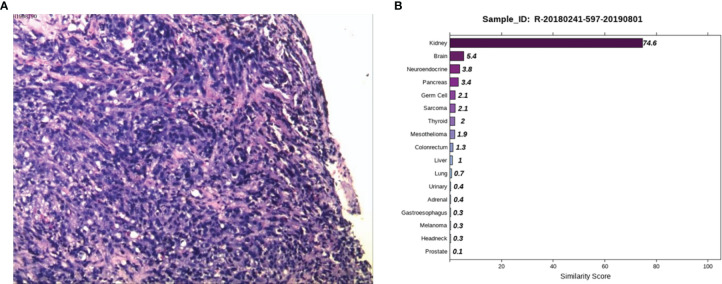
Pathological and 90-gene expression assay results of case 2. **(A)** The pathological results showed diffuse proliferation of spindle cells, atypical epithelioid cells in a sheet-like distribution, and necrosis and bone formation were seen. Immunohistochemical staining confirmed CK, LCK, Hep (focally), Urop3 nucleus as positive. **(B)** The 90-gene expression assay indicated that there is a 74.6 similarity score of metastatic renal cancer on the right side of the bone.

Case 2: A 59-year-old male appeared with lumbosacral pain accompanied by obvious traction pain of the right lower extremity, which was aggravated after sitting and activity, without symptoms such as coughing, sputum, chest tightness, night sweats, or fatigue. Positron emission tomography/computer tomography (PET/CT) performed in a local hospital showed small nodules in the posterior right upper lung with low glucose metabolism and multiple bone destruction throughout the body with increased glucose metabolism. Pathological examination of high-metabolism sites showed that the spindle cells had proliferated diffusely, the epithelioid cells were aberrant and distributed in a sheet, and necrosis and sequestrum formation were visible. Next, he was admitted to Hunan Cancer Hospital for further diagnosis and treatment. After examining the pathological section (**Figure 2A**), we considered it to be a poorly differentiated metastatic malignant tumor. Epithelioid differentiated sarcoma was not excluded because this sarcoma can appear in the form of sarcomatoid carcinoma with transparent cell cytoplasm. The IHC results showed CK (+), LCK (+), P40 (-), P63 (-), Hep (+, focally), ERG (-), CD31 (-), Syn (-), CgA (-), NapsinA (-), GATA3 (-), TTF1 (-), PSA (-), TG (-), and Urop3 nucleus (+). The enhanced CT scans of the neck, chest, abdomen, and pelvis showed multiple damaged areas of cervical, thoracic, lumbar vertebral, and right iliac bone, and no other malignant lesions in the lung, liver, kidney, and prostate. Brain magnetic resonance imaging (MRI) revealed the abnormal bone quality of the right frontal bone and C2 vertebra. To eliminate digestive tract tumors, gastroscopy and colonoscopy were performed and results showed no obvious abnormalities. To further identify the primary site, the 90-gene expression assay was performed on the biopsy tissue of the right iliac bone and the test results indicated that the right iliac bone had metastatic kidney cancer (**Figure 2B**). The patient was diagnosed with primary renal clear cell carcinoma (TxN0M1, stage IV). Then the patient returned to the local hospital for receiving palliative radiotherapy and chemotherapy.

**Figure 3:**
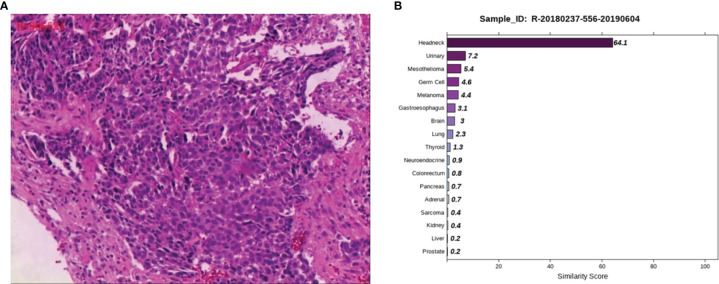
Pathological and 90-gene expression assay results of case 3. **(A)** The pathological diagnosis of the resected specimens was metastatic poorly differentiated carcinoma. Immunohistochemistry staining: CK-pan, Ki-67, P63, P40 were positive respectively. **(B)** The 90-gene expression assay indicated that the similarity score of bone biopsy tissue is head and neck tumor metastasis was 64.1.

Case 3: A 53-year-old male, appeared with numbness of the left upper limb without radiation pain and other discomforts such as dizziness, headache, nausea and vomiting. Lumbar MRI in a local hospital showed lumbar disc herniation. No therapeutic outcome was achieved in the patient after being firstly treated with unknown medication. The patient reported the worsened situation and then surgery was planned in a hospital in Hunan Province on May 7, 2019. The lumbar MRI before surgery showed a mass in the left front of S1, S2 vertebral with bone marrow edema, and the left nerve root was violated. They are highly suspicious to be tumors and a sacral puncture biopsy was performed on May 20, 2019. The histopathological findings (**Figure 3A**) demonstrated metastatic poorly differentiated carcinoma. The IHC results showed CK-pan (+), CK7 (-), CK20 (-), Villin (-), Ki-67 (70%+), P63 (+), P40 (+), Hepatocyte (-), Glypican-3 (-), PSA (-), PSAP (-), D2-40 (-), CR (-), and RCC (-). To find a primary tumor, PET/CT was performed in a hospital in Changsha city on May 30, 2019, found that the bone on the left side of S1 and S2 vertebrae was damaged with increased glucose metabolism, no obvious abnormality in the head, neck, chest and abdomen organs. The patient underwent colonoscopy, gastroscopy, and prostate color doppler ultrasound without any obvious abnormalities in our hospital on June 3, 2019, and the pelvic MRI revealed bone destruction and mass formation on the left side of the sacrum. Pathological diagnosis of the nasopharyngeal biopsy showed a large amount of inflammatory cell infiltrate in the mucosa, and atypical small squamous epithelial cells were seen in the interstitium. Epstein-Barr encoding region (EBER) *in-situ* hybridization showed negative results. To further clarify the primary tumor, the 90-gene expression assay was performed on the sacral biopsy tissue, and the test results indicated that the sacral biopsy tissue was head and neck tumor metastasis (**Figure 3B**). The patient’s head and face related MRI showed abnormalities in bilateral tonsils but he refused to undergo surgical biopsy of bilateral tonsils. We considered the possibility that the primary tumor is tonsil cancer and give systemic palliative chemotherapy (albumin paclitaxel and carboplatin) combined with local radiotherapy. The patient is stable now and has survived for more than two years.

**Figure 4:**
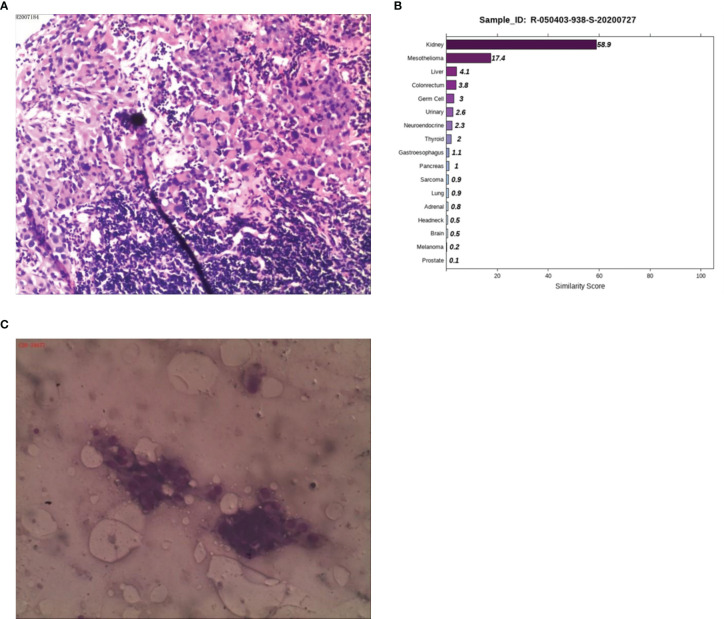
Pathological, 90-gene expression assay and cytology results of case 4. **(A)** The pathological diagnosis of the resected specimens was malignant tumors which tend to be metastatic poorly differentiated. Immunohistochemistry staining: CK (+++), CK7 (-), CK20 (-), Villin (-), TTF1 (-), SATB-2 (+), MelanA (+/-), NapsinA (-), P40 (-), CR (-), CK5/6 (-), CDX-2 (-), S-100 (-), Des (-), SOX-10 (-), HMB45 (-). **(B)** The 90-gene expression assay indicated that the similarity score of metastatic renal cancer and liver cancer on the left cervical lymph node was 58.9 and 17.4, respectively. **(C)** The cytology results showed cancer cells in the kidney puncture smear.

Case 4: A 50-year-old male presented with abdominal pain and discomfort without obvious inducement in May 2019. The pain was paroxysmal and dull, which accompanied by lower abdomen swelling. The pain was obvious when fasting, and it was relieved after eating. In June 2020, abdominal pain significantly worsened than before. CT examination at a local hospital showed multiple retroperitoneal lymphadenopathies, and B-ultrasonography showed a lymph node cyst in the left neck and supraclavicular fossa. A biopsy of the left cervical lymph node was performed on June 28, 2020. The postoperative examination revealed a malignant tumor in the left neck, and poorly differentiated squamous cell carcinoma was considered (**Figure 4A**). The patient visited Hunan Cancer Hospital on July 8, 2020 for further treatment. CT examination revealed multiple lymphadenopathies in the left supraclavicular area, mediastinum, hilum, retroperitoneum, and right iliac vessels. Head MRI examination showed no obvious occupying in the nasopharyngeal region. We consulted the local hospital regarding the pathological sections and combined IHC, and considered the diagnosis of malignant tumors, which tend to be poorly differentiated metastasis. Because IHC provides no clear indication, it is difficult to determine the tumor type. It is recommended to check the kidney, lung, liver, prostate, etc. in detail. IHC demonstrated a result of CK (+++), CK7 (-), CK20 (-), Villin (-), TTF1 (-), SATB-2 (+), MelanA (+/-), NapsinA (-), P40 (-), CR (-), CK5/6 (-), CDX-2 (-), S-100 (-), Des (-), SOX-10 (-), and HMB45 (-). PET-CT was performed to understand the general condition, results showed multiple swollen lymph nodes on the double supraclavicular, mediastinum, double lung hilum, right posterior of the right diaphragm, retroperitoneal area, and right iliac vessels, and abnormal radioactive concentration was seen in the corresponding positions, no obvious abnormality in the head, neck, chest and abdomen organs. To further clarify the source of the tumor, the 90-gene expression assay was performed, and the results suggested that the tumors on the left cervical lymph node maybe a metastasis from kidney tumors (**Figure 4B**). We then performed a renal MRI examination and found a nodule at the lower pole of the right kidney with a diameter of approximately 1.4 cm, considering the possibility of renal cancer. For further confirmation, a CT-guided kidney biopsy was performed on August 11, 2020. The cytological results showed cancer cells in the kidney puncture smear (**Figure 4C**), but no clear malignant tumor was found in the pathological section. Because the kidney mass was small (approximately 1 cm), the pathological diagnosis of renal biopsy still could not be further confirmed. Combined with the results of genetic testing and cytology, we considered the possibility of renal cancer, and the patient was treated with the targeted drug pazopanib. On September 18, 2020, the patient returned to the hospital for review. CT of the chest and abdomen showed a nodule with a density of 1.3×1.1 cm in the lower pole of the right kidney, which was smaller than before. At present, he is in stable condition and is undergoing immunotherapy.

## Discussion

Case 1 was a squamous cell carcinoma lesion in the lung found five years after surgery of cervical squamous cell carcinoma. The pathological test was not able to distinguish whether it was cervical cancer lung metastasis or second primary lung cancer. The 90-gene expression assay results suggested second primary cancer. Notably, treatment plans and prognoses differ completely between metastasis and second primary cancer. There is still the possibility of a cure through surgery if it is second primary cancer, while metastasis can only be treated with palliative care. The 90-gene expression assay helped distinguish second primary cancer from recurrent metastasis.

Case 2 involved bone metastases with an unknown primary site. There were no obvious lesions observed from the imaging examination. The 90-gene expression assay results suggested that the source of the tumor was the kidney. The 90-gene expression assay can facilitate the discovery of occult lesions and has guided the follow-up treatment.

Case 3 was a case of bone metastasis with no primary tumor. The imaging examination was normal. The 90-gene expression assay suggested that it was a head-and-neck tumor. Based on the 90-gene expression assay result, we performed an MRI of the head and face, with results suggesting the tumor site was tonsil. The 90-gene expression assay can quickly identify the primary site, thereby shortening the inspection time and reducing the opportunity cost of medical interventions.

Case 4 was a malignant tumor with multiple lymph node metastases; however, the immunohistochemistry results of left neck lymph node biopsy showed no clear source of tumor. The 90-gene expression assay results suggested that the source of the tumor was likely to be the kidney. Kidney biopsy cytology was performed to find cancer cells, but the pathological section could not identify malignant tumors. On August 1, 2020, the kidney MRI showed a nodule with a diameter of approximately 1.4 cm at the lower edge of the right kidney. After oral treatment with the kidney cancer-targeted drug pazopanib for a month, CT was performed, and the size of the right renal nodule was 1.3 x 1.1 cm, having diminished in size, suggesting that the treatment was effective. In the absence of clear and solid evidence from traditional diagnostic methods, the 90-gene expression assay can help to quickly identify suspicious target lesions, enabling patients to receive early and accurate treatment.

A so-called “primary tumor of unknown origin” is diagnosed partly because the primary tumor is too small to be found from imaging. The possible reasons are as follows: 1) the primary focus grows slowly because of the body’s strong immunity, 2) some cells are poorly differentiated, and distant metastasis appears at an early stage when the primary tumor is too small to be detected by traditional diagnostic methods, and 3) in a few patients, the primary tumor had been removed many years ago; the primary tumor disappeared, but distant metastasis appeared. Over the past decades, genomic profiles including gene expression profiling, DNA methylation and genomic alteration have been developed rapidly to identify tumor tissue of origin (6–8). The prognosis of a CUP patient largely depends on the biological characteristics of the primary tumor. Hainsworth et al. demonstrated that CUP patients who received a tumor type-specific therapy based on the results of gene expression profiling analysis had better median survival than CUP patients who received empiric therapy (12.5 *vs.* 9.1 months) (4). Therefore, clarifying the tissue origin of the tumor and adopting targeted treatment are of great significance for improving the prognosis of patients. Herein, the 90-gene expression assay can help elucidate the primary tumor source, and it may become a helpful molecular diagnostic method for CUP patients in the future.

## Data Availability Statement

The raw data supporting the conclusions of this article will be made available by the authors, without undue reservation.

## Ethics Statement

Written informed consent was obtained from the relevant individual(s), and/or minor(s)’ legal guardian/next of kin, for the publication of any potentially identifiable images or data included in this article.

## Author Contributions

XP: Data analysis and participation in the writing of the manuscript. SY: Data analysis and interpretation and participation in the writing of the manuscript. YX: Data analysis and interpretation. BC: Data analysis and interpretation. QW: Data analysis and interpretation. QG: Collection and assembly of data. LW: Study design and direction and writing of the first draft of the manuscript. All authors contributed to the article and approved the submitted version.

## Funding

This work was supported by Hunan Provincial Natural Science Foundation of China (No: 2019jj80018 to XP), Changsha Science and Technology Project of China (No: KJ1901073 to XP).

## Conflict of Interest

The authors declare that the research was conducted in the absence of any commercial or financial relationships that could be construed as a potential conflict of interest.

## Publisher’s Note

All claims expressed in this article are solely those of the authors and do not necessarily represent those of their affiliated organizations, or those of the publisher, the editors and the reviewers. Any product that may be evaluated in this article, or claim that may be made by its manufacturer, is not guaranteed or endorsed by the publisher.

## References

[1] Hasegawa H Ando M Yatabe Y Mitani S Honda K Masuishi T. Site-Specific Chemotherapy Based on Predicted Primary Site by Pathological Profile for Carcinoma of Unknown Primary Site. Clin Oncol (2018) 30:667–73. doi: 10.1016/j.clon.2018.06.012 30196846

[2] Pavlidis N Fizazi K. Cancer of Unknown Primary (CUP). Crit Rev Oncol/Hematol (2005) 54:243–50. doi: 10.1016/j.critrevonc.2004.10.002 15890271

[3] Varadhachary GR Raber MN. Cancer of Unknown Primary Site. N Engl J Med (2014) 371:757–65. doi: 10.1056/nejmra1303917 25140961

[4] Hainsworth JD Rubin MS Spigel DR Boccia RV Raby S Quinn R. Molecular Gene Expression Profiling to Predict the Tissue of Origin and Direct Site-Specific Therapy in Patients With Carcinoma of Unknown Primary Site: A Prospective Trial of the Sarah Cannon Research Institute. J Clin Oncol (2013) 31:217–23. doi: 10.1200/jco.2012.43.3755 23032625

[5] Richardson A Wagland R Foster R Symons J Davis C Boyland L. Uncertainty and Anxiety in the Cancer of Unknown Primary Patient Journey: A Multiperspective Qualitative Study. BMJ Support Palliat Care (2013) 5:366–72. doi: 10.1136/bmjspcare-2013-000482 24644189

[6] Ye Q Wang Q Qi P Chen J Sun Y Jin S. Development and Clinical Validation of a 90-Gene Expression Assay for Identifying Tumor Tissue Origin. J Mol Diagn (2020) 22:1139–50. doi: 10.1016/j.jmoldx.2020.06.005 32610162

[7] Moran S Martinez-Cardús A Sayols S Musulén E Balañá C Estival-Gonzalez A. Epigenetic Profiling to Classify Cancer of Unknown Primary: A Multicentre, Retrospective Analysis. Lancet Oncol (2016) 17(10):1386–95. doi: 10.1016/S1470-2045(16)30297-2 27575023

[8] Hayashi H Takiguchi Y Minami H Akiyoshi K Segawa Y Ueda H. Site-Specific and Targeted Therapy Based on Molecular Profiling by Next-Generation Sequencing for Cancer of Unknown Primary Site: A Nonrandomized Phase 2 Clinical Trial. JAMA Oncol (2020) 6(12):1931–8. doi: 10.1001/jamaoncol.2020.4643 PMC7563669 33057591

